# Supplementary material for: Exogenous Application of Low-Concentration Sugar Enhances Brassinosteroid Signaling for Skotomorphogenesis by Promoting BIN2 Degradation
Source: Int J Mol Sci. 2021 Dec 18;22(24):13588. doi: 10.3390/ijms222413588 (PMC8706690; doi:10.3390/ijms222413588)
Supplement: Supplementary file 1 [file ijms-22-13588-s001.zip › ijms-1485064-supplementary.pdf]

Supplementary Information

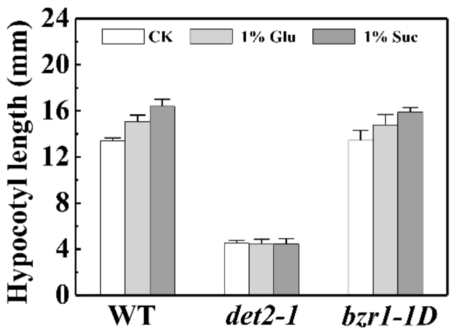

Figure S1. Hypocotyl length of WT, *det2-1* and *bzr1-1D* plants treated with glucose. Error bars represent SD (n > 18).

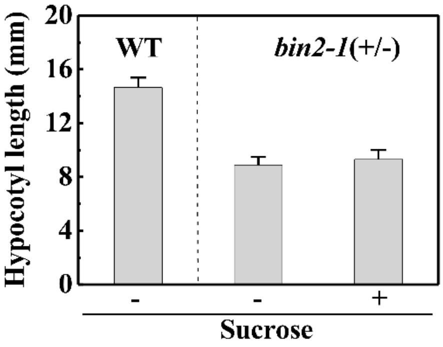

Figure S2. Heterozygous *bin2-1*(+/-) mutant in responsive to sucrose. Error bars represent SD (n > 25).

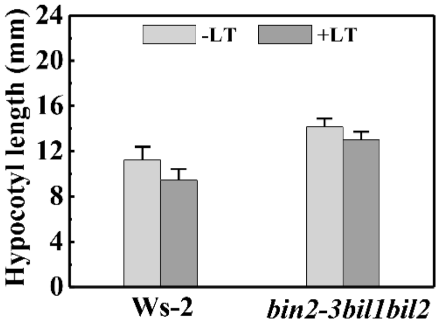

Figure S3. Hypocotyl length of Ws-2 and *bin2-3bil1bil2* plants treated with S6K inhibitor (LY2584702 Tosylate, LT). Error bars represent SD (n > 18).

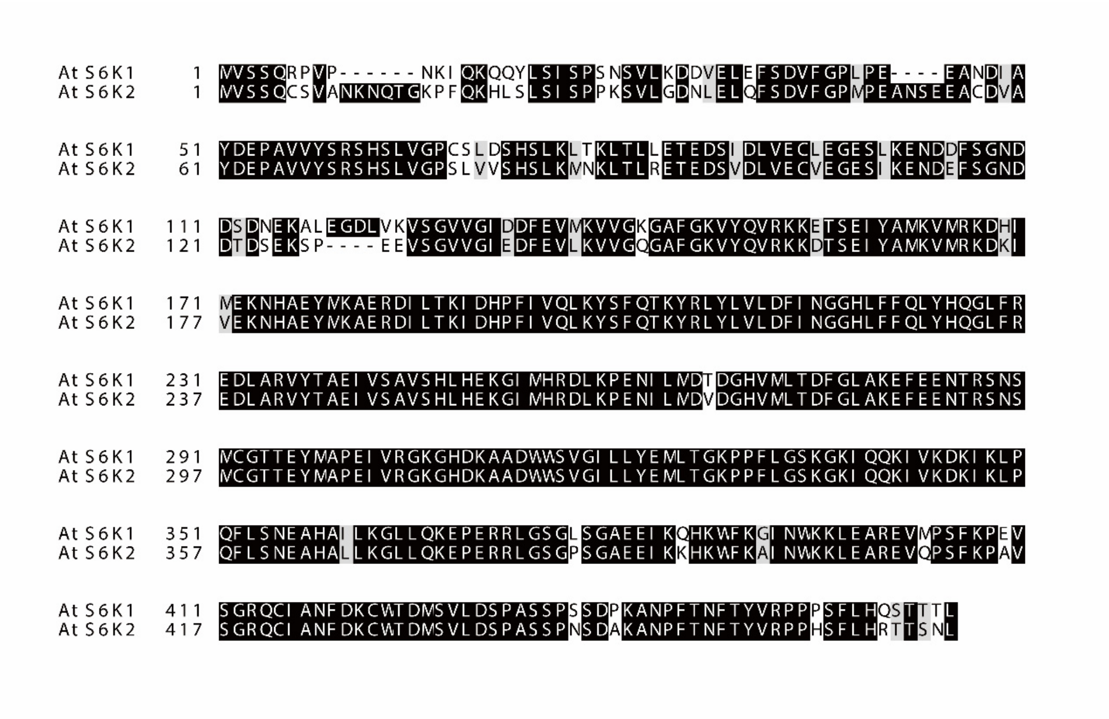

Figure S4. Amino acid sequence alignment of S6K1 and S6K2.

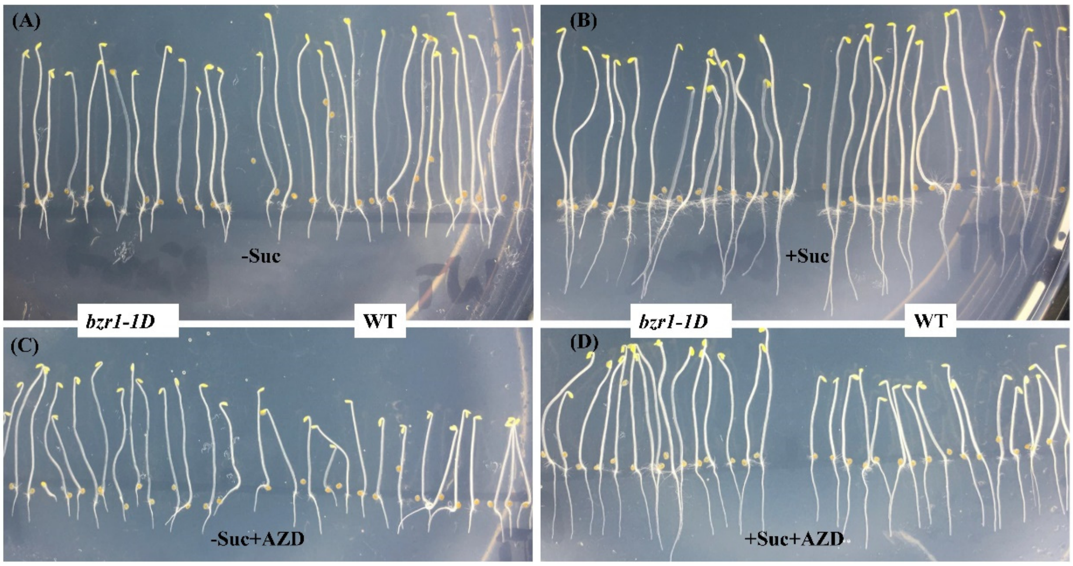

Figure S5. Root length of WT and *bzr1-1D* plants with different treatments.

Table S1. Primer sequences used for qRT-PCR.

BIN2-qF:<br>BIN2-qR:	ACAAAAGGATGCCCCCAGAA<br>TGAAGTTGAAGAGAGGCGGG	Li et al. (2020)
BZR1-qF:<br>BZR1-qR:	CAACTAGGCAAACCCAAATG<br>TCTAACACTCCAATGCTTCC	
BIL1-qF:<br>BIL1-qR:	AACTCGCGAAGAAATCCGGT<br>AAGGTCAATGGCTTCCGGAG	This study
BIL2-qF:<br>BIL2-qR:	TCCATTACCGCCATTGTTCA<br>TTCTCTTCAGACATGCACAG	Zhang et al. (2021)
UBQ10-qF:<br>UBQ10-qR:	AATTGGAGGATGGTCGTACTTT<br>CAAAGTCTTGACGAAGATCTGC	This study

Li, J.; Terzaghi, W.; Gong, Y.; Li, C.; Ling, J.J.; Fan, Y.; Qin, N.; Gong, X.; Zhu, D.; Deng, X.W. Modulation of BIN2 kinase activity by HY5 controls hypocotyl elongation in the light. *Nat. Commun.* **2020**, *11*, 1592. https://doi.org/10.1038/s41467-020-15394-7.

Zhang, W.; Tang, Y.; Hu, Y.; Yang, Y.; Cai, J.; Liu, H.; Zhang, C.; Liu, X.; Hou, X. Arabidopsis NF-YCs play dual roles in repressing brassinosteroid biosynthesis and signaling during light-regulated hypocotyl elongation. *Plant Cell* **2021**. https://doi.org/10.1093/plcell/koab112.
